# Lipid-Coated Mesoporous Silica Particles for pH-Sensitive Tumor-Targeted Paclitaxel: Development, Characterization

**DOI:** 10.7150/jca.117433

**Published:** 2025-08-22

**Authors:** Yingyue Deng, Tao Zhang, Jiaru Zhou, Zhihao Su, Yingsong Cao, Junxi Luo, Leming Zhao, Junjie Hua, Guoqiang Wang, Min Xiao, Junfeng Ban, Yan Zhang, Hongcai Liang

**Affiliations:** 1 Guangdong Pharmaceutical University; Guangzhou, China.; 2 The Innovation Team for Integrating Pharmacy with Entrepreneurship, Guangdong Pharmaceutical University, Guangzhou, China.; 3 Guangdong Provincial Hospital of Chinese Medicine, The Second Affiliated Hospital of Guangzhou University of Chinese Medicine, Guangdong Provincial Academy of Chinese Medical Sciences, Guangzhou, Guangdong 510006, China.

**Keywords:** Paclitaxel, mesoporous silica nanoparticles, liposomes, antitumor, pH sensitivity, controllable drug delivery

## Abstract

Nanoparticle carriers can selectively deliver the drug cargo to tumor cells, thus having the ability to prevent early drug release, reduce non-specific cell binding, and prolong *in vivo* drug retention. We constructed paclitaxel (PTX)-loaded lipid-shell mesoporous silica nanoparticles (LMSNs) for targeted anti-cancer drug delivery. The physical properties of PTX-LMSNs were analyzed by scanning electron microscopy (SEM) and transmission electron microscopy (TEM). The drug loading *(DL*%) and entrapment efficiency (*EE*%) of PTX-LMSNs were measured by high performance liquid chromatography (HPLC). *In vitro* drug release test, *in vivo* imaging, tissue distribution and pharmacokinetics of PTX-LMSNs were also evaluated. The SEM examination showed that MSNs were sphere, whereas TEM showed that they were rich in fine pores. The uniform core-shell structure of PTX-LMSNs was also verified by TEM. The DL capacity of PTX-LMSN was as high as 21.75%, and PTX was released from the nanoparticles *in vitro* in a pH-dependent manner. The cumulative amount of free PTX increased at lower pH, which is conducive to selective drug release from LMSNs in the acidic tumor tissues. *In vivo* imaging showed prolonged retention of PTX-LMSNs, which is beneficial to their therapeutic efficacy. In addition, PTX-LMSNs were primarily concentrated in the liver. Pharmacokinetic experiments showed that the half-life of PTX-LMSNs was 23.21% longer and 79.24% higher than that of Taxol. Together, LMSNs are a highly promising antineoplastic drug carrier system.

## Introduction

Chemotherapy is still an indispensable tool for treating advanced cancer. However, the inability of traditional anti-cancer drugs to specifically target tumor cells not only leads to serious systemic side effects but also limits their therapeutic effects. In recent years, nanoparticle drug delivery systems, such as liposomes, polymeric micelles, and multimers, have attracted extensive research attention in the field of antitumor drug carriers due to their unique drug loading capacity, targeted delivery characteristics, and controllable release performance 1-3. Nanoscale carriers achieve passive tumor targeting by enhancing the osmotic retention effect (EPR), significantly increasing the accumulation concentration of drugs in tumor tissues, and improving the solubility and stability of hydrophobic drugs 4, 5. However, traditional carriers prepared by nano-preparations such as liposomes are unstable in body fluids and also release the drug cargo before reaching the tumor, resulting in serious adverse effects 6, 7.

Particle size is an important parameter for efficient delivery of nanocarriers to tumor sites, as well as for their high adsorption on the tumor cell surface, cellular uptake and intracellular transport, which eventually determine the efficacy of chemotherapy 8-10. Stimuli-responsive drug delivery systems dynamically adjust the particle size in response to changes in pH, enzyme, heat or magnetic field of the tumor microenvironment, prolong the cycle and enhance the EPR effect in the early stage, and promote tumor penetration in the later stage, which can significantly improve the ability to penetrate deep tumor tissues, thereby optimizing the efficiency of drug delivery and balancing the contradiction between permeability and retention 11-13. Therefore, stimuli-responsive drug delivery systems can enhance the targeted accumulation of drugs at the lesion site, thereby improving the therapeutic effect and possibly reducing damage to normal tissues 14-16. Stimulus-responsive drug delivery systems are designed to respond to a variety of physical and chemical stimuli such as temperature, electric field, pH, magnetic field, and ionic strength, with pH and temperature being widely used because they do not require additional lasers and cause less damage to normal tissues 17.

Mesoporous silica is biodegradable, and the degradation product, silicic acid, can be absorbed and excreted through the urinary system. Lee 18 reported that 0.1 mg/mL mesoporous silica nanoparticles (MSNs) could be completely degraded within 7 days in simulated body fluids *in vitro*. Although mesoporous silica nanoparticles (MSNs) have good biocompatibility, they still have two major limitations in practical applications: first, the drug is easy to leak in advance during delivery, and it is difficult to achieve precise targeted and controlled release; Second, the uptake efficiency is low in some specific cell types, such as primary cells and non-phagocytic cells, which directly affects the delivery and therapeutic efficacy of the drug. These deficiencies limit the wide application of MSN in clinical treatment to a certain extent 19. Nevertheless, owing to their high surface area, pore volume, uniformity, biocompatibility and biodegradability, MSNs are highly promising inorganic drug carriers 20-22.

Lipid-shell and mesoporous silica core nanoparticles (LMSNs) combine the characteristics of nanoparticles and liposomes. The dual vesicular and particulate structure is associated with high biocompatibility, stability and favorable pharmacokinetic profile 23, 24. Furthermore, drugs can be efficiently encapsulated within the polymer core or between the lipid bilayers of LMSNs, thus allowing LMSNs to have high loading capacity 25. The polymer core might also delay drug diffusion and increase the stability of the lipid shell, thereby enhancing the encapsulation efficiency (EE) and system stability 26. In contrast, polymer micelles are relatively less stable because they are thermodynamic self-assembled structures formed by reversible stabilizing forces such as hydrophobic effects and electrostatic interactions, which will inevitably be disintegrated by multiple instability mechanisms in complex *in vivo* environments, resulting in drug leakage, protein adsorption, and dilution below critical micelle concentrations 27. However, it is challenging to achieve high EE and optimal particle size when LMSNs is incorporated into hydrophilic drugs. To this end, we are committed to the development of a high-efficiency paclitaxel (PTX) delivery system based on mesoporous silica nanoparticles (LMSNs), PTX-LMSNs. PTX-LMSNs makes full use of the advantages of high drug loading capacity and small particle size of mesoporous silica nanoparticles, and significantly improves the encapsulation efficiency and tumor targeting of the drug through careful surface modification and stimulus response design, effectively overcoming the defects of traditional polymer micelle drugs that are easy to leak and insufficient targeting, thereby greatly enhancing the anti-tumor effect and treatment accuracy of paclitaxel. LMSNs were prepared using a modified method, and PTX-LMSNs were comprehensively characterized. The distribution of the nanovehicles in mice was observed by real time *in vivo* imaging. The *in vivo* pharmacokinetics of the drug was also evaluated.

## Materials and Methods

### Materials

Paclitaxel (PTX) was purchased from Hainan Yayuan Pharmaceutical Co. Ltd. (Hainan, China), ethyl orthosilicate (TEOS) was from Aladdin Chemical Reagent Co. Ltd. (Shanghai, China), cetyltrimethyl ammonium bromide (CTAB) was from McLean Chemical Reagent Co. Ltd. (Shanghai, China), NaHCO_3_ was from Tianjin Zhiyuan Chemical Reagent Factory (Tianjin, China), and 15-hydroxy stearic acid polyethylene glycol (Solutol HS-15) was from BASF Company. Egg yolk lecithin (PL100M) was purchased from Shanghai AVT Pharmaceutical Technology Co. Ltd. (Shanghai, China). Other chemicals and solvents were of analytical grade.

### Synthesis of mesoporous silica core-lipid bilayer shell nanoparticles

The preparation of MSNs started by dissolving 1.5 g of CTAB and K_2_SO_4_ in a 255 ml solution consisting of 5 mL of 95% ethanol and 0.5 M NaHCO_3_ ammonia (solution 1). Solution 2 was then prepared by mixing 5 mL of 0.5 M Ca(COO)_2_ in 5 mL of 95% ethanol. The two solutions were quickly mixed with 10 mL of TEOS and then stirred at 600 r/min at room temperature for 6 h. After aging for 4 h, the reaction products were washed thrice with 75% ethanol at 4000 r/min, purified and then dried in vacuum at 65 ºC. The dried mesoporous silica carrier was soaked in 0.1 M hydrochloric acid, washed thrice with 75% ethanol at 4000 r/min, and then dried in vacuum at 65 ºC. Subsequently, it was calcined in a muffle furnace at 250 ºC for 0.5 h and then heated to 550 ºC for 4 h to obtain MSNs.

The description of lipid-modified MSNs is shown in Table 1, and the lipid-modified MSNs were prepared by post-loading method 28, 29. Briefly, 63 mg of PTX was dissolved in 5 volumes of dichloromethane in a penicillin vial, and 3-fold MSNs were then added. The mixture was stirred at 500 r/min for 4 h in the dark at room temperature. After removing the organic solvents, 50 mL of aqueous solution containing 0.5% Solutol HS-15 was poured into a round-bottomed flask, and lecithin and oleic acid were added thereafter using the membrane dispersion method. The lipids were dissolved in 25 mL of trichloromethane, and the solvent was evaporated at 40 ºC until a uniform and transparent lipid film was formed. The MSN preparation was suspended in a hydration solution, and the resultant solution was poured over the lipid coating and then swirled until the lipid membrane was completely dissolved 30. After that, it was continuously sonicated at 100 W for 5 min using an ultrasonic processor before being filtered through a 0.22 μm nylon microporous membrane. The filtrate was then added with a small volume of ampoule while passing nitrogen, and the obtained PTX-LMSNs were stored at 4 ºC.

### Characterization of nanoparticles

The morphology of MSNs was observed by scanning electron microscopy (SEM, JSM-7500F, JEOL, Japan) and transmission electron microscopy (TEM, JEM-1400, JEOL, Japan), and the morphology of PTX-LMSNs was observed by TEM. MSNs were gilded before being observed under a scanning electron microscope. The MSNs were dispersed in deionized water to a concentration of 2 mg/mL prior to TEM examination, while the PTX-LMSNs were diluted by 10 times. The particle size and polydispersion index (PDI) of PTX-LMSNs were measured by a Delsa Nano C (Beckman Coulter, USA), and their morphology was observed under a transmission electron microscope. Nitrogen adsorption-desorption was analyzed by a JW-BK 132F specific surface area and porosity analyzer (Beijing, China). The specific surface area was calculated by Brunauere-Emmette-Teller (BET) method, and the cumulative pore volume was calculated by Barrett-Joyner-Halenda (BJH) model.

### Drug content and encapsulation efficiency (EE%)

The drug loading (*DL*%) capacity of PTX-LMSNs was measured by high performance liquid chromatography (HPLC, 1260, Agilent Technologies, USA) using a Kromasil C18 (4.6 mm × 250 mm, 5 μm) column. The mobile phase consisted of methanol, acetonitrile and distilled water. The flow rate was 1 mL, the injection volume was 20 μL and the detection wavelength was 229 nm. To extract PTX, 1 mL of PTX-LMSN was mixed with 9 mL of acetonitrile, and the solution was filtered through a 0.22 μm membrane. The filtrate was injected into the HPLC instrument to determine the *DL* of PTX-LMSNs. PTX-LMSNs were extracted and separated by dextran chromatography, followed by HPLC to determine the entrapment efficiency *(EE*%). The SephadexG-50 separation column (1.5 cm × 13 cm) was equilibrated for 12 h before being loaded with 0.5 mL of PTX-LMSNs. The flow rate of the eluant was 3 mL/min, and the eluent was collected every 2 mL. After eluting with 30 mL of distilled water, the appropriate amount of eluent was collected and then mixed with 3 mL of acetonitrile to determine the amount of encapsulated drug (*W_en_*). Thereafter, 30 mL of 5% sodium dodecyl sulfate (5%, v/w) was used to elute free PTX (*W_free_*). The *EE*% 31 was calculated using the following formula:


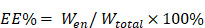
 (1)

### *In vitro* drug release assay

*In vitro* drug release was measured by the dialysis method. Briefly, 1 mL of PTX-LMSNs solution was loaded into a prepreg dialysis bag (molecular weight cut-off = 8000-14000). The dialysis bag was then immersed in 20 mL of buffer containing 1% sodium dodecyl sulfate with pH = 5, 6.8 or 7.4 at a constant temperature of 37 ºC while gently shaken at 100 r/min. A 2 mL aliquot of sample was withdrawn at 0, 1, 2, 4, 8, 12, 24, 48, 72, 96 and 120 h and subjected to HPLC analysis. The withdrawn sample was replaced with the same volume of fresh buffer. The experiment was repeated three times. The cumulative release rate (*Q*) of PTX was calculated using the following formula:


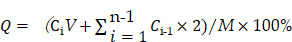
 (2)

where *C*_i_ is the sample concentration at the time point i,* V* is the volume of the suspension plus the release medium in the dialysis bag, and* M* is the dose of PTX in the dialysis bag.

### *In vivo* biodistribution

Comply with the ARRIVE guidelines and were carried out in accordance with the U.K. Animals (Scientific Procedures) Act, 1986 and associated guidelines, and EU Directive 2010/63/EU for animal experiments. C57 Mice were depilated and anesthetized by intraperitoneal injection with 1.3 g/kg uratan solution (> 98%, 65mg/mL), and injected intravenously with 0.1 mL of fluorescent Rhodamine-LMSNs. The mice were imaged at 5, 30, 120 and 360 min post-injection and euthanized for 6 h after imaging. The heart, liver, spleen, lungs and kidneys were dissected, washed with deionized water and placed in a Petri dish. The tissues were imaged at an excitation wavelength of 550 nm and an emission wavelength of 600 nm for a fixed exposure time of 0.1 s. After the experiment, the mice were euthanized by cervical spondylolysis.

### Pharmacokinetics analysis

Animal experiments have been approved by the Institutional Animal Care and Use Committee of Guangdong Pharmaceutical University and comply with the ARRIVE guidelines and were carried out in accordance with the U.K. Animals (Scientific Procedures) Act, 1986 and associated guidelines, and EU Directive 2010/63/EU for animal experiments. SD Rats were fasted and weighed 12 h before the experiment. The animals were randomly divided into two groups (6 per group, equal number of male and female) and injected with 20 mg/kg PTX-LMSNs via the tail vein. Blood samples (250 µL) were collected via the retroorbital route at 0, 0.083, 0.5, 1, 2, 3, 4, 6, 8 and 12 h post-injection and placed in heparin-coated tubes. After the experiment, rats were euthanized by inhaling CO_2_.The blood samples were centrifuged at 3000 r/min for 10 min, and the upper plasma layer was collected. The plasma (100 μL) was mixed with an equal volume of acetonitrile, vortexed for 1 min, sonicated for 1 min (40 kHz, 300 W), and then centrifuged at 10000 r/min for 10 min. The samples were then analyzed by HPLC.

### Statistical analysis

SPSS17.0 statistical software was used for statistical analysis. The data were expressed as mean ± standard deviation (SD). Comparison of the tested samples with the control samples was carried out by analysis of variance and t-test. P < 0.05 was considered statistically significant.

## Results

### Characterization of MSNs and PTX-LMSNs

**Figure 1:**
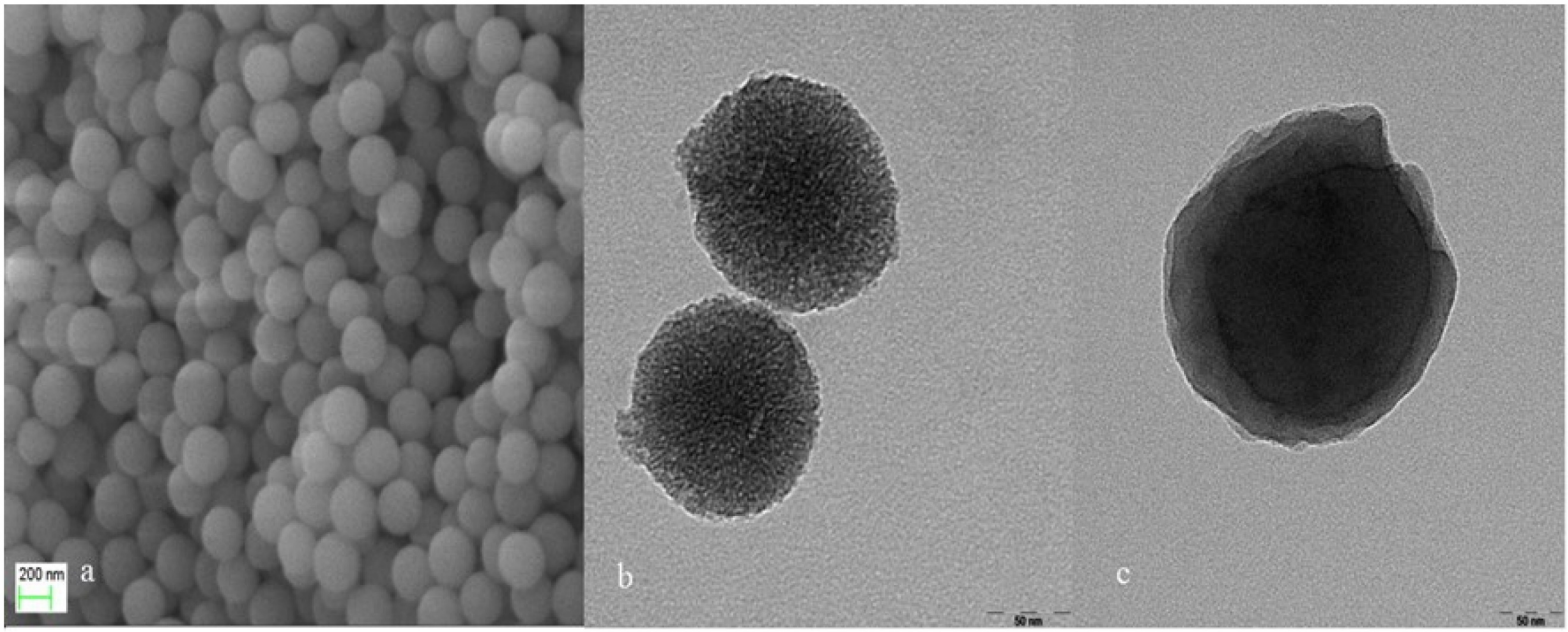
(a) SEM image of MSNs. (b) TEM image of PTX-MSNs. (c) TEM image of PTX-LMSNs.

MSNs were morphologically characterized by SEM and TEM. As shown in the SEM images in Figure 1a, the MSNs had a uniform and regular spherical structure. Furthermore, TEM analysis verified the presence of pores on the MSNs, as indicated by the contrasting dark pore wall and bright channel in the images shown in Figure 1b. The particle diameter is about 150 nm. In addition, the pore walls appeared distinct and were densely arranged. TEM examination of the PTX-LMSNs (Figure 1c) revealed that they were spherical particles with a uniformly thick surface lipid layer and a distinct core-shell layer structure.

**Figure 2:**
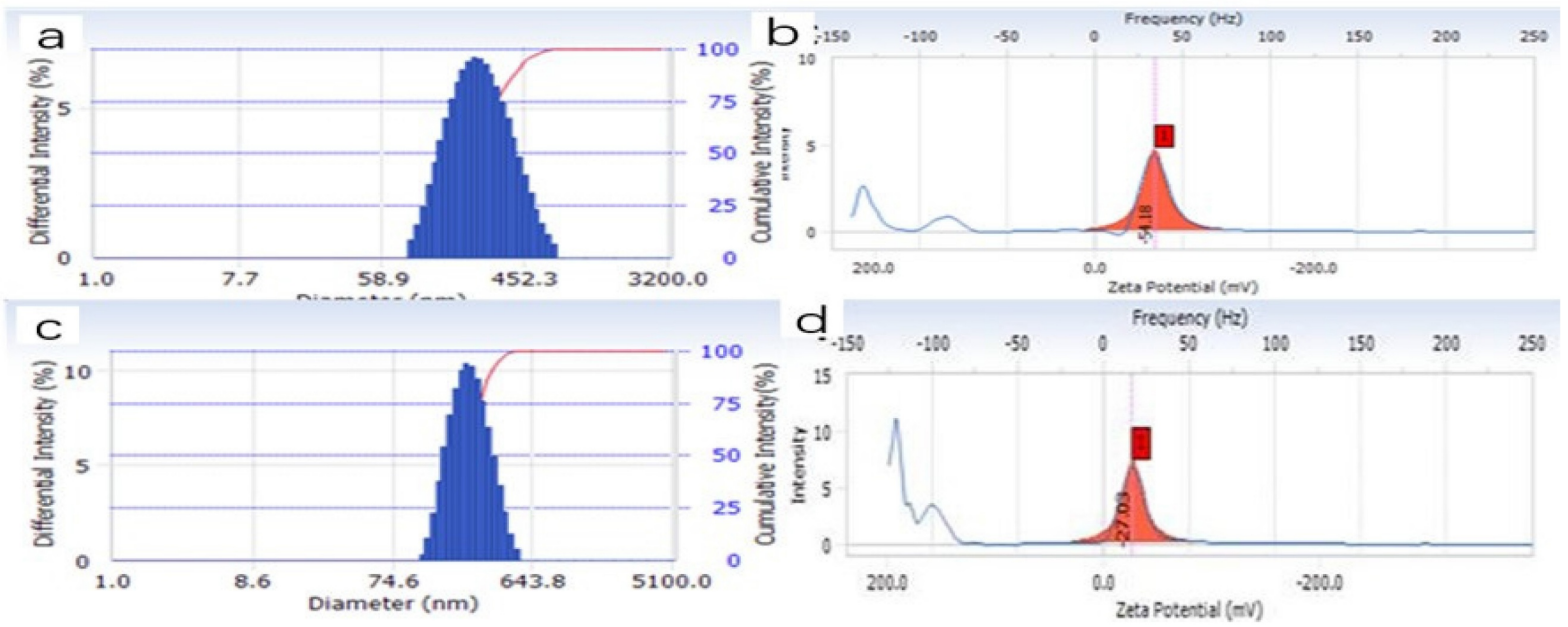
Particle size distribution and zeta potential of MSNs. (a) Particle size distribution of PTX-LMSNs. (b) Zeta potential of MSNs. (c) Particle size distribution of PTX-LMSNs. (d) Zeta potential of PTX-LMSNs.

As shown in Figure 2a, the average particle size of MSNs was 225.6 ± 9.44 nm, and their PDI was 0.097 ± 0.02. These numbers indicate that MSNs have high stability and uniform particle size distribution. Furthermore, MSNs had a negative charge, according to their Zeta potential (Figure 2b). PTX-LMSNs also had a uniform particle size distribution, as indicated by the single symmetrical peak (Figure 2c). The average particle size and PDI of PTX-LMSNs were 245.8 ± 3.26 nm and 0.102 ± 0.02, respectively. Although the particle size of MSNs increased as a result of lipid modification, it remained at around 220 nm. In addition, the lipid shell had no effect on PDI, indicating that the incorporation of phospholipids did not interfere with the stability of the MSNs. As shown in Figure 2d, the lipid modification also did not cause a significant change in Zeta potential, which remained in a range of -15 to -30 mV.

According to the IUPAC nomenclature, the adsorption of MSNs belongs to a typical type IV isotherm 32. The pore size distribution of MSN was narrow, and the average pore size was about 3.05 ± 0.04 nm, which is a suitable size allow the incorporation of PTX.

### Drug loading and encapsulation efficiency (EE) of PTX-LSNs

The DL and EE of PTX-LMSNs were 21.75 ± 4.28% and 96.36 ± 1.93%, respectively. This may be due to the fact that most drug molecules were loaded into the mesoporous silica pores, and only a few of them were adsorbed on the outer surface; these may cause a small amount of drug to dissociate into the solution during the preparation of lipid layer. The high drug loading and high encapsulation efficiency of PTX-LMSNs make them have significant clinical translational value, which can improve the therapeutic effect of paclitaxel and reduce the toxicity and side effects, providing a new strategy for the clinical application of tumor targeted therapy.

### *In vitro* release of PTX from PTX-LMSNs

**Figure 3:**
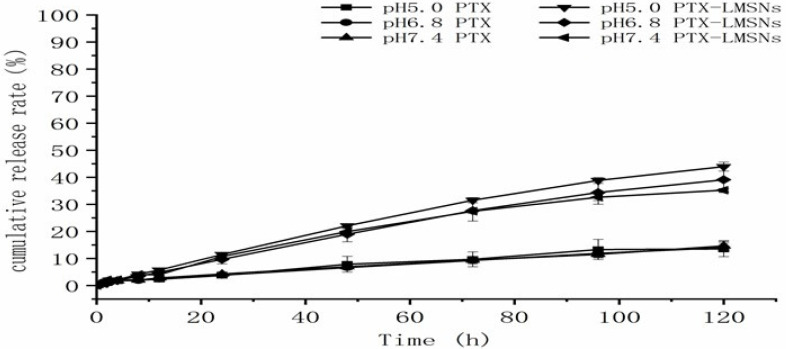
*In vitro* release profile of PTX from PTX-LMSNs under different pH conditions.

Unlike weakly alkaline healthy tissues, tumors have a low pH due to the Warburg effect wherein the increase of oxygen consumption leads to the production of acidic metabolites 33. Therefore, we exposed PTX-MSNs and PTX-LMSNs to different pH levels that simulate the pH of normal tissues (pH 7.4), extracellular fluid of tumor tissues (pH 6.8) and the endosomes or lysosomes (pH 5), and then measured the amount of drug released at 37 ºC. As shown in Figure 3, the cumulative amount of PTX released from the LMSNs was 3-4 times higher than that released from the unmodified MSNs at all tested pH conditions, which is an indication sustained drug release. Furthermore, the cumulative amount of drug released per unit time from PTX-LMSNs was highest at pH 5;11.48% of the encapsulated drug was released within 24 h, 22.07% within 48 h, and 43.98% within 5 days. This can be attributed to the instability of phospholipid membranes under acidic conditions 34. Protons in an acidicc medium can accelerate the replacement of the drug, thus leading to its rapid release. Therefore, the LMSN carrier is pH-sensitive and can selectively release the drug cargo at the tumor site. The cumulative increase of PTX in the acidic tumor tissues can not only improve the efficacy of the drug but also minimize the damage to normal tissues and cells.

### Biodistribution of antitumor drug in mice

**Figure 4:**
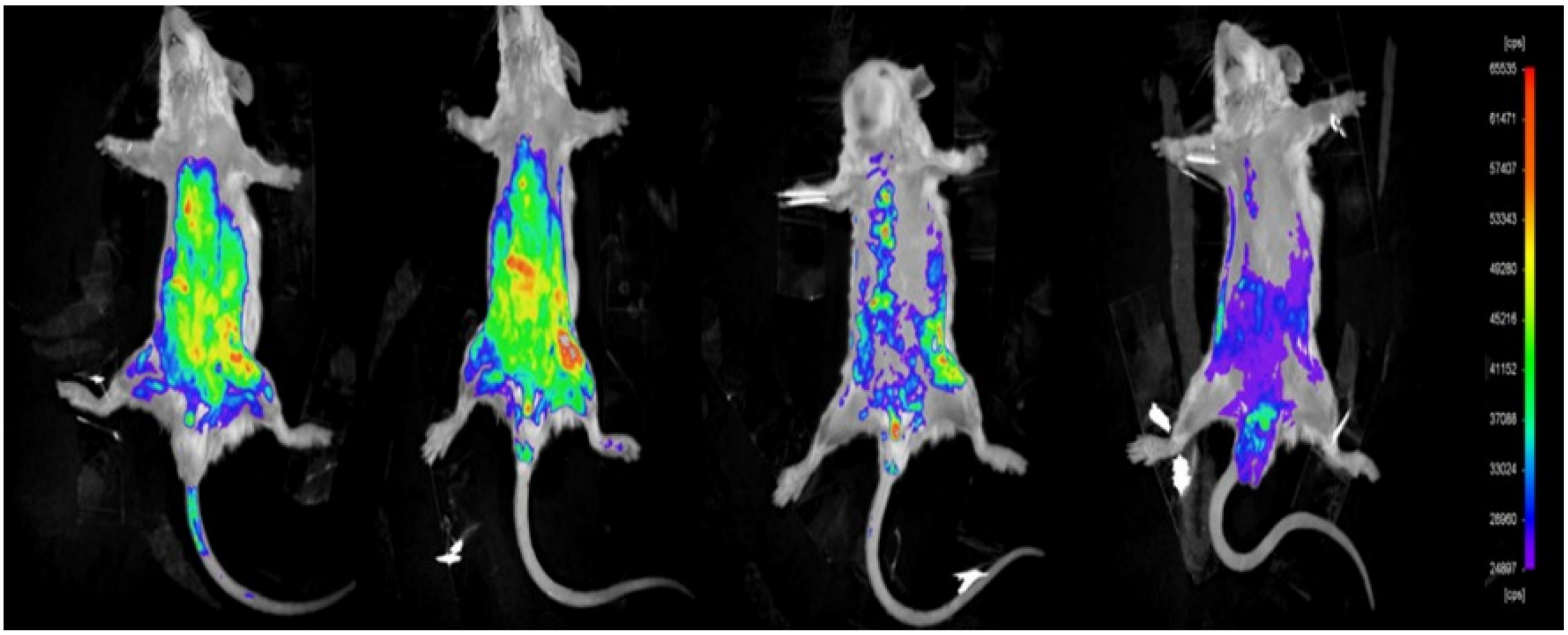
Distribution and fluorescence intensity of PTX-LMSNs probe in mice.

Distribution and fluorescence intensity of PTX-LMSNs in mice are illustrated in Figure 4. The probe entered the body circulation within 5 min of injection, and the fluorescence intensity of PTX-LMSN in the abdomen increased with time and was especially high in the liver and spleen. In addition, the strong fluorescence in the bladder may be related to drug metabolism. The fluorescence intensity of PTX-LMSNs in the bladder decreased after 2 h of administration. After 6 h, fluorescence was nearly undetectable in any of the organs.

**Figure 5:**
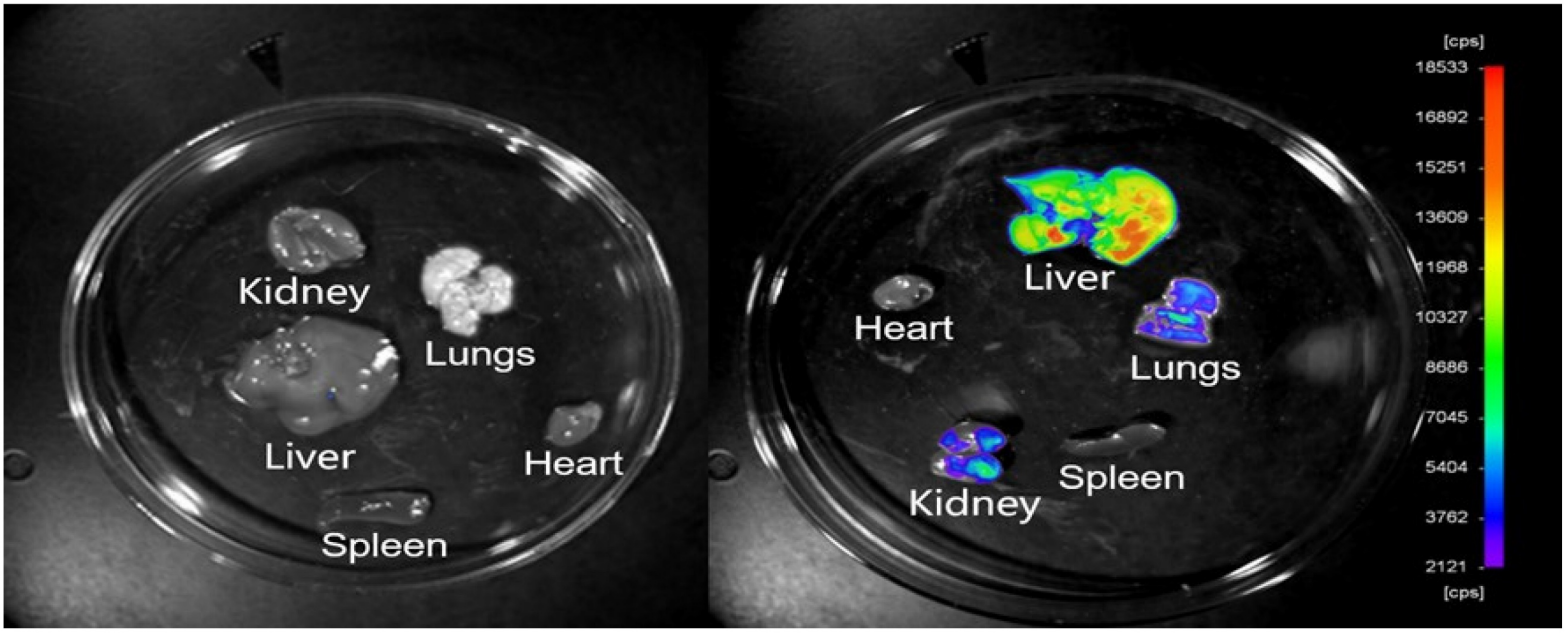
Tissue distribution of fluorescent PTX-LMSN (a: control; b: PTX-LMSNs)

Organs were removed 6 h after administering with the PTX-LMSNs and subjected to *ex vivo* fluorescence imaging. As shown in Figure 5, the drug mainly accumulated in the liver, which could be due to the abundance of macrophages that can recognize opsins (serum proteins) adsorbed on the surface of the nanoparticles. Hepatic accumulation enhances the anti-tumor effect by increasing the local concentration of paclitaxel at the site of liver tumors while reducing systemic distribution to minimize side effects; however, excessive accumulation may increase the metabolic burden on the liver, leading to drug-induced liver injury or diminished efficacy against metastasis to other organs. Modifying dosage forms and using combination therapy strategies can maximize benefits and reduce risks.

**Figure 6:**
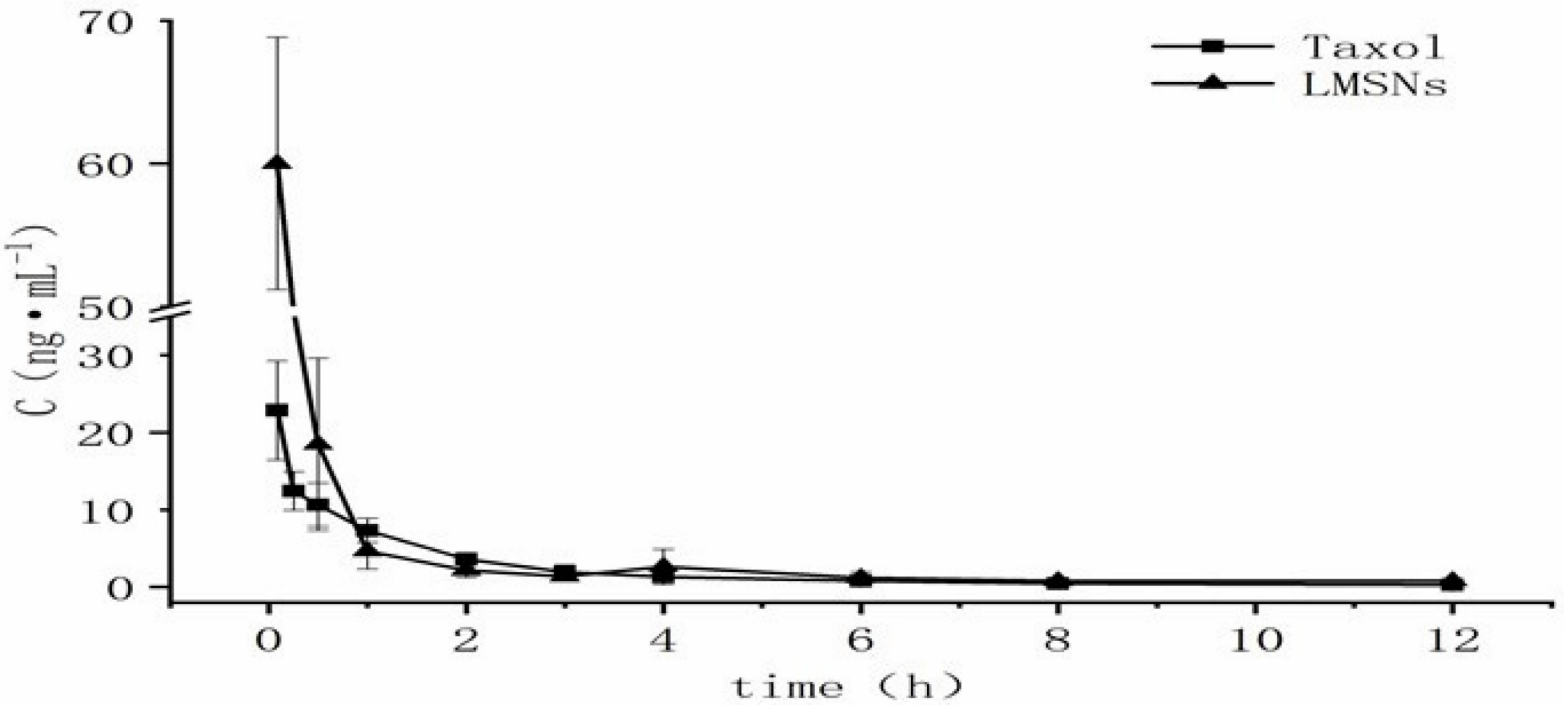
Concentration-time curve.

The pharmacokinetic parameters of Taxol and the PTX-LMSNs were fitted by DAS software with the two-compartment model and are summarized in Table 2. As shown in Figure 6, the *in vivo* half-life of PTX-LMSNs was 23.21% higher than that of Taxol, and the area under the curve (AUC) was twice as high, an indication of longer drug retention, higher therapeutic efficacy and lower toxicity.

## Conclusions

We successfully prepared PTX-LMSNs by fusing liposomes with MSNs. PTX-MSNs were sensitive to low pH, making them ideal drug carriers for the acidic TME, as they could be controlled to release the drug cargo in a pH-responsive manner. The targeted delivery of the drug cargo to the tumor site improves therapeutic efficacy while minimizing damage to normal tissues. Real-time *in vivo* near-infrared fluorescence imaging showed a fairly board large size distribution range of PTX-LMSNs, which may be related to their low metabolism. Visceral fluorescence intensity map further indicated that PTX-LMSNs were mainly clustered in the liver. This can be attributed to the fact that the abundant macrophages in the liver can recognize opsins (serum proteins) adsorbed on the surface of the nanoparticles. *In vivo* pharmacokinetics showed that PTX-LMSNs had a higher AUC compared to that of Taxol, indicating that they have longer retention. This property is beneficial for high drug efficacy and low toxicity. PTX-LMSNs exhibit high drug loading, small particle size, and pH-responsive drug release, which have significant advantages in preclinical studies. Subsequently, it is necessary to promote its transformation into clinical trials through system toxicology evaluation, process scale-up and optimization, and targeting efficiency optimization. This study provides a new strategy for the development of nanocarrier-based tumor targeted delivery systems, but it is necessary to further address the issues of large-scale production and long-term safety.

## Figures and Tables

**Table 1:** The prescription of lipid modification of mesoporous silica nanoparticles.

Component	Content
Paclitaxel	1.10%
MSN	3.30%
Oleic acid	0.29%
Soybean Phospholipid	2.90%
Solutol HS-15	0.48%
Trichloromethane	q.s.
Dichloromethane	q.s.
Water	Add up to 100%

**Table 2:** Pharmacokinetic parameters in mice (n=5).

Parameters	Taxol	LMSNs
t1/2α/h	0.056	0.069
t_1/2β_/h	1.208	0.287
Vd/L*Kg-1	234.833	155.146
K21/h-1	4.201	5.109
K10/h-1	1.476	3.241
K12/h-1	7.363	4.157
AUC/mg·L-1·h	28.848	51.707
CL/L·h-1·kg	346.642	502.835

## Abbreviations

PTX: paclitaxel
MSNs: Mesoporous silica nanoparticles
LMSNs: lipid-shell mesoporous silica nanoparticles
PTX-LMSNs: paclitaxel-loaded lipid-shell mesoporous silica nanoparticles
SEM: Scanning electron microscopy
TEM: Transmission electron microscopy
DL%: The Drug loading
EE%: Entrapment efficiency
HPLC: High performance liquid chromatography
TEOS: Ethyl orthosilicate
CTAB: Cetyltrimethyl ammonium bromide
Solutol: HS-15 15-hydroxy Stearic acid polyethylene glycol
PDI: Polydispersion index
BET: Brunauere-Emmette-Teller
BJH: Barrett-Joyner-Halenda
SD: Standard deviation
AUC: Area under the curve
